# Graphemic Analysis and the Spoken Language Bias

**DOI:** 10.3389/fpsyg.2016.00388

**Published:** 2016-03-21

**Authors:** Kristian Berg

**Affiliations:** Institut für Germanistik, Carl von Ossietzky Universität OldenburgOldenburg, Germany

**Keywords:** graphemic analysis, spoken language, correspondences, writing system, phonocentrism

In the history of linguistics, the investigation of written language got off on the wrong foot. For a long time, writing was regarded as a secondary medium, its raison d'être being the recording of spoken language (cf. e.g., de Saussure, 1916/1959, p. 23f.; Bloomfield, 1933, p. 500ff.). Spoken language in turn was regarded as the primary object of linguistic investigation. Consequently, there was no interest in an unbiased analysis of writing; if writing was analyzed at all, it was seen through the eyes of phonology^1^.

It took decades for things to improve, but written language was eventually accepted as a linguistic object in its own right. Yet the old spoken-language-bias is still influential, for example in the call for papers to this research topic. It is (as mostly nowadays) stated implicitly, which makes it harder to tackle. The topic editors state the importance of prosody for spoken language and the lack of its explicit marking in written language. On this basis they suggest that “the informativeness of written text may seem astonishing.” This line of reasoning could be called the *phonocentric fallacy*: What is important in spoken language must be important in written language; moreover, there cannot be independent structures and relations in writing mediating between graphemic form and meaning, i.e., bypassing the phonological route.

In the following, I briefly will show the main shortcomings of phonocentric approaches to writing. In doing so, I will use examples from this research topic's call for papers—not because the editors stand out with their opinion, but precisely because they do not. The phonocentrism manifested in the call for papers is shared in a number of current papers on writing. Note that I do not disagree with many questions raised in the call—they are justified and interesting. It is the general perspective that is biased and that I feel should be more balanced. As an alternative to this position, I will outline a theoretical framework that is descriptively more adequate and that (as a consequence) does writing more justice. Most of what follows is neither new nor original (cf. e.g., Vachek, 1939; Hjelmslev, 1943/1961; Venezky, 1970; Eisenberg, 1983; Cummings, 1988). It has been said many times before, often under the rallying cry of the “autonomy” of writing systems. However, I feel that this research topic calls for some specific comments, and I am grateful to the topic editors for giving me the possibility to express them.

Phonocentric approaches in their strongest form take written language to be secondary to and derived from spoken language. From this follows that it is futile to investigate writing autonomously: All graphemic units and relations are reducible to phonological units and relations, and basic principles of theory building (such as Ockham's Razor) prohibit unnecessary theoretical entities^2^. Punctuation, for example, is regarded as a means of marking intonation (cf. call for papers). However, the derivative nature of writing should be a hypothesis, not an axiom. It may turn out to be true (although there is much evidence against it, see below) or false; the crucial point is that its status can only be determined on the basis of an unbiased analysis (cf. Eisenberg, 1988).

As indicated above, the phonocentric view on writing is old. At its core probably lies a mix-up of arguments (cf. Eisenberg, 2013, p. 286): The derived nature of writing may be very plausible diachronically—spoken language is older than written language. But as Stetter (1997, p. 62) observed, the constitutional principle of alphabetic writing (one spoken segment corresponds to one written segment) and its functional principle are two very different things. Just because alphabetic writing probably evolved as phonographic writing does not mean it exclusively serves that function after (in the case of languages like English, French, and German) centuries of use. What is more, the nature of this use shifted from reading aloud to silent reading in the late Middle Ages (cf. Saenger, 1997). In silent reading, we can (in principle) directly access the semantic side of written words, without taking the phonological “detour.”

But, one might ask, what practical harm is done in regarding punctuation as a marking of intonation? None, in principle, and as I will argue below, the closely related question of *how intonation is marked in spelling* is valid and interesting. What is potentially harmful to the description of writing, however, is the common presupposition that all of writing, all units and relations, are determined by phonological units and relations. As many abstract theoretical issues, this one too has very practical consequences: Phonocentric approaches cannot account for “non-phonographic” spellings—spellings that cannot be captured in phonological terms. I will demonstrate just two such cases in the following; for convenience sake, I will use examples from English.

First, there are constraints on word-final letters, for example on <v> (cf. e.g., Venezky, 1999, p. 83ff.). Consider the following words:

(1) have, give, live, dative, evolve, groove

Phonographically, these words might as well be spelled with final <v> (^*^ <hav>, ^*^ <giv>, ^*^ <liv>), but they are not. As a matter of fact, there is only a handful words with final <v> in English, most of them shortenings or acronyms (*derv, lav, rev*) or slang words (*spiv*). The final <e> in (1) cannot be explained with reference to phonology: It is purely graphemically motivated—it prevents word-final <v>.

Second, it can be observed that English derivational suffixes are often spelled in a distinct way that sets them apart from homophonous word endings (cf. Berg and Aronoff, accepted). Consider the words in (2) and (3). All of them end phonetically in [ɨs] (cf. Flemming and Johnson, 2007), at least in connected speech.

(2) nervous, hazardous, famous
(3) service, bonus, tennis

What sets both groups apart is that only adjectives are spelled with final <ous>, and that (with very few exceptions) *all* adjectives with final [ɨs] are spelled with <ous>. There is thus a very tight correlation between spelling and morphology: Spelling marks a morphological category, it makes morphology visible (cf. for similar phenomena in German Fuhrhop, 2011). Again, this phenomenon cannot be reduced to phonology. It is cases like this and many related ones that add to the “informativeness of written text” without providing phonological information.

So the phonocentric view on writing is inadequate: It fails to capture non-phonographic structures because it does not expect them. What is the alternative? Treat writing as a system in its own right. Try to uncover its structures and regularities. They come in two kinds:

1. Purely graphemic units and relations can be determined without reference to other representational levels like phonology or morphology. For example, writing—especially print—almost naturally breaks down into letters and words: There are spaces between words, and there are smaller spaces between letters. Letters and (graphemic) words are thus genuine graphemic units. Another example is the constraint on word final letters shown above. The categories “vowel” and “consonant” can also be established on graphemic grounds alone, using distributional methods (cf. Berg, 2012). This is not the case for, say, phonemes: The set of units that correspond to e.g., /s/ (<s>, <ss>, <ce> etc.) is not purely graphemic. The elements can only be grouped together because they correspond to one phoneme. Consider a parallel case in phonology: No one would seriously advocate that “subject,” “stem,” or “nominative” are phonological categories. They belong to different levels of representation, which should be kept apart analytically. The same holds for the analysis of writing: Genuine graphemic units are those that can be determined on graphemic grounds alone, not units superimposed on writing from other levels.
2. But these latter, superimposed units and relations are of course also relevant in the description of writing; they can be termed *morphographic, phonographic*, etc. Here, we leave the realm of pure graphemics, and we search for correspondences between writing and other representational levels: How consistently are affixes and stems spelled, and do these spellings in turn refer to affixes and stems exclusively (cf. e.g., Berg et al., 2014; Berg and Aronoff, accepted)? What corresponds to syntactic words? “How is prosody encoded in writing?” (call for papers). How is intonation marked in writing? All these questions are valid and interesting. In each instance, we take a non-graphemic unit (e.g., the suffix*-ous*, the phonological trochee) and analyze the set of corresponding spellings. How consistently is the respective unit spelled? How many exceptions are there? How consistently is the reverse mapping direction—e.g., are *all* and *only* trochees spelled in a specific way?

This can also be used to categorize writing systems typologically. How many purely graphemic units and relations are there? Are the phonographic or the morphographic correspondences more coherent, i.e., does the writing system lean toward representing phonological or morphological units, and if so, which units predominantly?^3^

One last comment regarding psycholinguistic evidence, as in e.g., the sophisticated study of Kentner (2012), which shows that readers are sensitive to prosody even in silent reading. What relevance do psycholinguistic data have on a model of the writing system? To my mind, both issues should be kept apart: As a linguist (and a structuralist aficionado), I conceive of the language system as abstract and non-mentalistic; it is embodied in a corpus of utterances. In a second step, we can ask how speakers, hearers, readers, and writers use this system. But the fact that readers even in silent reading probably build up prosodic structures (or the whole issue of subvocalization, for that matter) has no bearing on the language system as an abstract entity: It does not necessarily follow, for example, that our model of writing should include (grapho-) prosodic units. Of course, they should be incorporated into a model of the reading process. But the only criterion that is sufficient for the inclusion of such units in the writing system is if we can determine them autonomously, i.e., purely graphemically.

## Author contributions

The author confirms being the sole contributor of this work and approved it for publication.

### Conflict of interest statement

The author declares that the research was conducted in the absence of any commercial or financial relationships that could be construed as a potential conflict of interest. The reviewer MB and handling Editor declared their shared affiliation, and the handling Editor states that the process nevertheless met the standards of a fair and objective review.

## Footnotes

^1^There were of course many legitimate grounds for placing spoken language on a pedestal, the most interesting of which may have been the idea that all languages are equal, which is the cornerstone of any scientific study of language (thanks to Mark Aronoff for this comment).

^2^See Spade (2008) and Aronoff (2012) for a critical assessment of this principle though.

^3^In a way, this is a refined version of Frost and Katz's (1992) idea of “orthographic depth.”
